# Sensitivity towards Fear of Electric Shock in Passive Threat Situations

**DOI:** 10.1371/journal.pone.0120989

**Published:** 2015-03-27

**Authors:** Patrick Ring, Christian Kaernbach

**Affiliations:** 1 Kiel Institute for the World Economy, Kiel, Germany; 2 Department of Psychology, University of Kiel, Kiel, Germany; Centre national de la recherche scientifique, FRANCE

## Abstract

Human judgment and decision-making (JDM) requires an assessment of different choice options. While traditional theories of choice argue that cognitive processes are the main driver to reach a decision, growing evidence highlights the importance of emotion in decision-making. Following these findings, it appears relevant to understand how individuals asses the attractiveness or riskiness of a situation in terms of emotional processes. The following study aims at a better understanding of the psychophysiological mechanisms underlying threat sensitivity by measuring skin conductance responses (SCRs) in passive threat situations. While previous studies demonstrate the role of magnitude on emotional body reactions preceding an outcome, this study focuses on probability. In order to analyze emotional body reactions preceding negative events with varying probability of occurrence, we have our participants play a two-stage card game. The first stage of the card game reveals the probability of receiving an unpleasant electric shock. The second stage applies the electric shock with the previously announced probability. For the analysis, we focus on the time interval between the first and second stage. We observe a linear relation between SCRs in anticipation of receiving an electric shock and shock probability. This finding indicates that SCRs are able to code the likelihood of negative events. We outline how this coding function of SCRs during the anticipation of negative events might add to an understanding of human JDM.

## Introduction

The evaluation of future outcomes is essential for human JDM. Traditional economic theory assumes that overt reasoning based on declarative knowledge is fundamental for this process [1]. Recent evidence, however, highlights a strong involvement of emotional processes [2–4]. Damasio [5], for example, argues that body signals—such as heart rate or skin conductance (SC)—guide human behavior before conscious knowledge does. According to his hypothesis, these so-called somatic markers fulfill an important role in decision-making. While negative somatic markers function as alarms for individuals to avoid an outcome, positive markers function as incentives for individuals to achieve an outcome. Somatic markers develop associative links between similar situations and past experiences. For example, touching a hot stove and getting burned leads to a negative emotional state due to feeling pain. The association between the circumstances and the following emotional reaction is internalized by the individual. If the individual is in a similar situation, the association is remembered and may therefore help to avoid the negative consequences of getting burned again [6].

Empirical support for Damasio’s somatic marker hypothesis (SMH) comes from studies using the Iowa Gambling Task (IGT). In the IGT, patients with damage to the ventromedial sector of prefrontal cortices and healthy control subjects choose one card from four available decks. While two decks result in higher immediate gains, but also higher losses, the other two decks result in lower immediate gains, but also lower losses. The gains and losses are balanced so that drawing cards from the first two decks leads to a net loss. Drawing cards from the other two decks leads to a net gain. In the course of the experiment, healthy participants adapt their decision-making strategies accordingly. Simultaneously, they produce anticipatory SCRs preceding disadvantageous decisions *before* they are able to verbally express the rule underlying this task. Patients, by contrast, do not follow an optimal strategy and also lack the above mentioned body reactions. However, some of them are able to verbally identify the advantageous decks at the end of the experiment. This indicates that, in these cases, overt reasoning might not be enough to make advantageous decisions. Missing body reactions in patients were interpreted as *insensitivity* towards future consequences, leading to impaired decision-making strategies [1], [7–9].

Decision problems in the IGT, as well as in the real world, are characterized by uncertainty. This means that particular decisions do not lead to certain outcomes, but varying outcomes with different probabilities are possible. Therefore, theories of JDM traditionally emphasize the role of both magnitude and probability of future outcomes on decision-making [10]. Following evidence that stresses the role of emotion in JDM, such as the SMH, it appears reasonable to assume that individuals show some sensitivity in terms of emotional reactions towards both components of a decision problem. This sensitivity should express itself as a change in the somatic state that is triggered by a stimulus giving information about future outcomes. Without this sensitivity, it would be unclear how choice options could be assessed in terms of emotional processes.

A previous study by Bowers [11] potentially demonstrates the existence of this sensitivity. The author shows that participants receiving high electric shocks were significantly more aroused in terms of higher SCRs preceeding shock realization than participants receiving low electric shocks. Thus, Bowers identifies one aspect of the above mentioned sensitivity, i.e. the sensitivity towards the magnitude of negative events. Similar patterns were also observed with monetary stimuli. Studer and Clark [12] report greater SCRs during the selection of bets with higher possible losses. Therefore, participants seem to be sensitive towards the magnitude of both negative physical and negative monetary stimuli.

Although several studies point out sensitivity towards the magnitude of negative events, evidence on sensitivity towards the likelihood of negative events is controversial. Bankart and Elliot [13] analyze SCRs in anticipation of shocks with varying probability of occurrence. In their study, 40 male undergraduates were assigned to one of four groups. Each group was given 8 shocks in 8, 11, 16 or 32 trials. By distributing shocks over a larger number of trials, the probability of receiving an electric shock in each single trial supposedly decreases. The authors did not find shock probability effects in terms of SCRs in the four groups. In studies by Epstein and Roupenian [14], Curtis et al. [15] and Mead and Dengerink [16] different groups of participants are given different information regarding the likelihood of receiving an electric shock. While the first study finds the greatest reactions in terms of SCRs in the group with a 5% shock probability, the latter two studies find the highest SCRs in the group with a 90% shock probability. Chandrasekhar et al. [17] find linear increasing SCRs during the anticipation of electric shock with a 0%, 33%, 66% and 100% probability of occurrence. Studer and Clark [12] indicate a sensitivity of body reactions preceding monetary outcomes with different probabilities. The authors report different patterns of heart rate responses during the selection of monetary bets with lower and higher chances of winning. SCRs, in their study, were not sensitive towards this aspect of a choice problem. Altogether results are controversial regarding the existence of a sensitivity towards the probability of negative events in terms of SCRs.

Based on recent findings regarding the role of emotion in JDM, sensitivity in terms of emotional body reactions towards the magnitude and probability of negative events seems relevant for the individual assessment of a situation. Due to controversial findings in the literature, this study focuses on emotional body reactions in terms of SCRs preceding negative events with a varying probability of occurrence in a within-participant design. Negative events were realized by means of electric shock. The choice of electric shocks instead of monetary stimuli is due to general problems of simulating monetary losses in experimental setups. For a further discussion on this topic, see [18]. We analyze emotional body reactions preceding negative events with varying probability of occurrence, while our participants play a two-stage card game. The first stage of the card game reveals the probability of receiving an unpleasant electric shock. The information about the shock probability is encoded as a combination of card value and condition under which a participant wins or loses a card bet. The second stage realizes the electric shock with the previously announced probability. For the analysis we focus on the 7 seconds (s) time interval between the first and the second stage. Our main finding identifies a positive graded relation between the likelihood of receiving an electric shock and SCRs during the anticipation phase, indicating a potential coding of probability.

## Materials and Methods

### Participants and Confederate

Thirty-two right-handed undergraduate psychology students (gender: 21 female, 11 male; age: *M* = 23.3 years, *SD* = 4.7) took part in our study in exchange for course credit points. All participants gave written informed consent and could decide to discontinue participation at any time. The research design was approved by the Ethics Committee of the German Psychological Society and performed in agreement with the Declaration of Helsinki.

### Experimental Design

**Fig 1:**
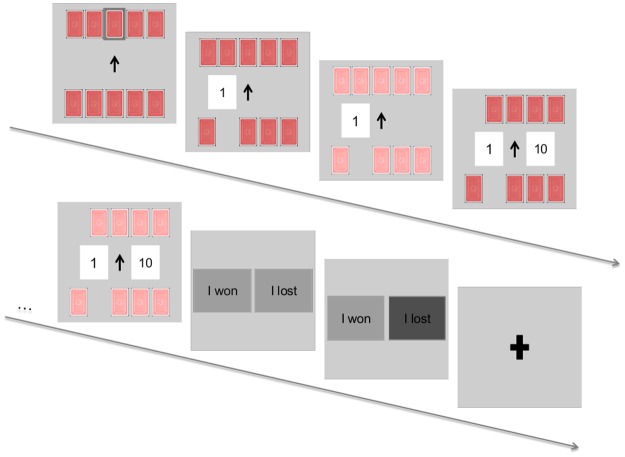
Experimental design.

Each participant played the following card game with the computer. First, the participant sees a screen with ten cards face down and receives the information that each card has a number from 1 to 10 and each number exists only once. Then, the participant draws one card. While choosing a card, an arrow in the middle of the screen indicates whether the higher or the lower card wins. Once the participant has chosen a card, that card is immediately turned over. Finally, a computer-drawn card from the remaining nine follows 7 s later (Fig. 1). While winning has no consequences, losing leads to an electric shock with a 50% probability. This design is related to Preuschoff et al. [19]. We modified the general procedure with respect to two different aspects. Firstly, instead of betting on a higher or lower second card with both cards drawn by the computer, our participants choose the first card out of ten themselves under a given bet. This modification was made to keep motivation levels sufficiently high by giving participants more freedom and the option to develop hypotheses about any existent general rules (which did not exist). Secondly, instead of losing or winning money, participants received electric shocks if they lost a bet. The idea of giving a shock only in some of the lost cases is due to a general habituation effect. As we work on the fear of electric shock rather than on the shock itself, we decided that the shock would be given with a 50% probability. The type of the bet changes in each round.

This approach, which excludes any form of active decision-making processes in the period after the first card, enables us to analyze pure sensitivity towards the likelihood of unpleasant events which is not contaminated by active decision-making processes. Showing the first card implicitly reveals the probability of receiving an electric shock associated with this card. After revealing the first card, we measure SCRs until the second card is turned over. In order to ensure that participants pay attention, we subsequently ask them to indicate whether they won or lost the bet. Incorrect answers were also followed by an electric shock. The software package Psychtoolbox-3 (www.psychtoolbox.org) running on MATLAB 7.1 (MathWorks Inc., United States) was used for stimulus presentation and response acquisition.

Participants were firstly instructed in written form and then verbally. In addition, each participant performed a practice run of 2 rounds without electric stimulation, in order to understand the task instructions. Afterwards, each participant played a total of 60 rounds with electric stimulation. In order to cover the full range of possible cases, the sequence of the first cards was arranged in a way that allowed each possible outcome (combination of the bet type and the first card) to occur exactly six times in one session. The order of this sequence and the value of the second card were random. The average duration of each session was 45 minutes (min).

### Electric Shock Stimulation

Pain was delivered via electric stimulation with a Canicom 800 (Num’Axes, France) (Levels of stimulation: 15; frequency: 1.023 Hz; duration: 115 ms, power range: 0.5 to 206 mW). The stimulation was administered via two flat AG-AGCL electrodes of 10 mm diameter being placed at the medial phalanges of the digits II and III of the dominant hand. Individual levels of pain stimulation were calibrated using a standard procedure: Shocks were presented in an ascending series of intensity until the participant indicated that the shock was painful. Once a painful level was reached, the previous stimulation level was used during the experiment.

### Psychophysiological Measurements

We used a 16-channel bioamplifier (Nexus-16; Mind Media B.V., the Netherlands) and the corresponding recording software Biotrace (Mind Media B.V.) to record electrodermal responses. Unfiltered SC data were acquired using a customer-specific SC sensor. The sensor maintained a voltage of less than 0.8V between the two flat AG-AGCL 10 mm diameter electrodes, which were placed at the medial phalanges of the digits II and III of the non-dominant hand. In accordance with common recomendations [20], the electrode sites were prepared with an isotonic paste (TD-246, Discount Disposables) and there was a 5 min pause between attaching the electrodes and starting recording. SC data were sampled at 32 Hz.

### Data Analysis

Skin conductance data was analyzed using Ledalab (www.ledalab.de) applying continuous decomposition analysis to disentangle phasic components from tonic activity [21]. The integrated skin conductance response (ISCR), which is defined as the time integral of the phasic driver for a relevant time interval, was used as a measure for the phasic electrodermal response to a given stimulus. In order to account for the typical skewed distribution of magnitude of electrodermal responses, individual ISCRs were standardized by the formula: *ISCR* = *log*(1 + ∣*ISCR*∣) × *sign*(*ISCR*) [22], [23].

**Fig 2:**
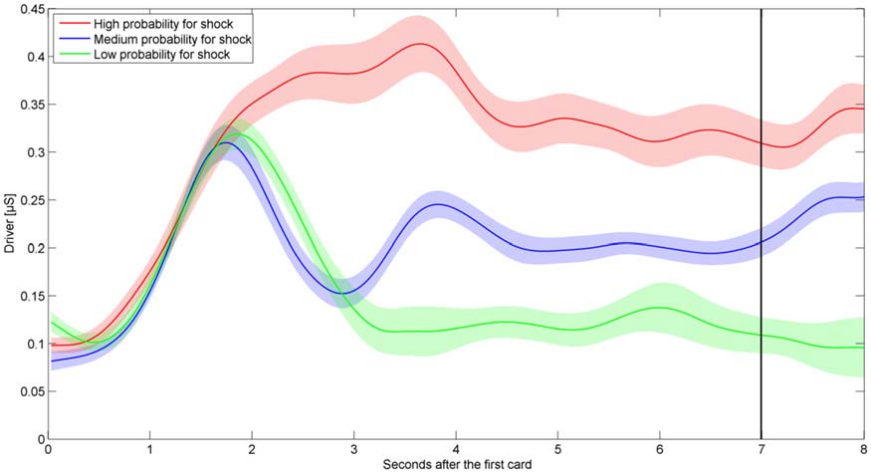
Phasic (driver) information after the first card for three cases (8 s). Shaded areas indicate the within-participant standard errors of the mean.

ISCRs for the time interval of 1-3 s after flipping the second card were used to analyze the effect of pain stimulation. The delay in SCRs has been reported in the literature [24]. In order to measure sensitivity towards the likelihood of receiving an electric shock, ISCRs were computed for the 5 s window starting 2 s after turning the first card. We skip the first two seconds in order to discard the effect of motor activation due to card selection (compare Fig. 2).

For ANOVA analyses, degrees of freedom were corrected by means of the Greenhouse-Geisser method were necessary and Bonferroni post-hoc tests were used for pair-wise comparisons of means.

## Results

### Task Performance

Participants, on average, won 49.27% (*SD* = 3.57%) of all trials. The mean time a participant needed to decide on one of the ten cards was 3.66 s (*SD* = 1.4 s) and to answer the control question was 1.23 s (*SD* = 0.51 s). Participants incorrectly reported the outcome of their bet in 0.57% (*SD* = 0.91%) of all cases.

### Effects of Electric Shock Stimulation

The average stimulation level was 9.38 (*SD* = 1.77) from 15 available levels. Repeated-measures ANOVA showed a significant difference between ISCRs after a won bet (*M* = 0.51 Log microsiemens (*μ*S) × s, *SE* = 0.02 Log *μ*S × s), a lost bet without shock (*M* = 0.82 Log *μ*S × s, *SE* = 0.02 Log *μ*S × s) and a lost bet with shock (*M* = 1.36 Log *μ*S × s, *SE* = 0.02 Log *μ*S × s), [Greenhouse-Geisser (G-G) corrected (*ɛ* = 0.72)], *F*(1.44, 44.73) = 156.1, *p* < 0.01. Bonferroni post-hoc tests showed significant differences between all three groups (*p* < 0.01). On a scale from 1 to 5 (not noticed (1), hardly noticed (2), acceptable (3), unpleasant (4), painful (5)), the average individual evaluation of stimulation after the experiment was 3.75 (*SD* = 0.44) and no participant indicated a pain level of 5 or less than 3.

### Effects of Electric Shocks with Different Probability of Occurrence

**Fig 3:**
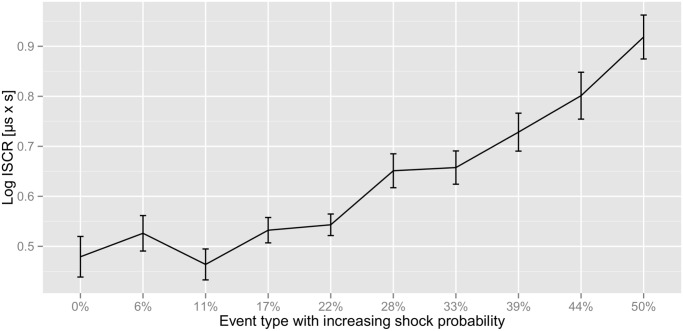
Average ISCRs for different event types with increasing shock probability. Error bars indicate the within-participant standard errors of the mean.

Fig. 2 shows the phasic (driver) information for 7 s after seeing the first card clustered into three shock probability groups (high/medium/low). The phasic driver increases in the first 2 s for all three groups due to motor activation, but splits afterwards. We observe a higher phasic driver in the high shock probability group compared to the low and medium shock probability groups. Similarly, the phasic driver of the medium shock probability group appeared to be higher than of the low shock probability group. Results were confirmed by statistical analysis. ISCRs after seeing the first card revealed a significant effect for the probability of receiving an electric shock [G-G corrected (*ɛ* = 0.49)], *F*(4.38, 135.87) = 17.5, *p* < 0.01. Further analysis showed a linear trend, *F*(1, 31) = 41.68, *p* < 0.01, with increasing probability of receiving an electric shock (Fig. 3).

## Discussion

In this study, we analyze how individuals assess the riskiness of a situation in terms of their emotional body reactions measured by SCRs. We are able to identify a mechanism that codes the probability for negative events. This mechanism appears relevant for an understanding of human JDM, because expectations regarding the riskiness of choice options affects decision-making. For example, the fear of an accident has an impact on the individual decision to fly or drive, even if this fear is not supported by objective measures [25]. Similarly, loss aversion, i.e. the fear of losing money, impacts investment decisions [26]. Moreover, fight-or-flight responses are classical examples for the role of emotion in facilitating action. In theses cases, potentially threatening events (e.g. a predator) alter the physiological state of individuals to support an action (i.e. the possibility to escape quickly) [27], [28]. In our experimental design, the threat is induced by electric shocks with different probabilities. Although the event per se is unavoidable (at least not without interrupting the experiment), we are able to observe changes in the somatic state of our participants preceding its realization. This result is discussed in the following part of the paper. The first part sets our result into perspective to previous research. We outline potential explanations for the controversial findings in the literature regarding the existence of a sensitivity towards probability in terms of SCRs. The second part concerns limitations of our study and possible follow-up studies. Finally, we provide more information on the single SCRs within the period of interest.

Studer and Clark [12] did not find effects of SCRs preceding monetary losses with different probabilities. Monetary losses, as stated in [18], are difficult to simulate in the laboratory environment. This potentially explains why the authors did not find any effects in terms of SCRs. The studies by Bankart and Elliot [13], Epstein and Roupenian [14], Curtis et al. [15] and Mead and Dengerink [16] find controversial results applying between-participant designs, i.e. each participant is only exposed to one probability of receiving an electric shock. Hence, the subjective evaluations of events might overweight the objective ones. For example, participants could think that rare events must be more severe than more common events. Therefore, framing effects might be an explanation for the divergent findings. Our results are in line with the study by Chandrasekhar [17], which finds linear increasing SCRs during the anticipation of electric shock with a 0%, 33%, 66% and 100% probability of occurrence. The authors also applied a within-participant design. In this study, two out of the four analyzed cases do not involve any uncertainty, but they are deterministic (0% and 100%). Risky situations in the real world, however, typically contain uncertainty and are rarely deterministic. Our design allows us to analyze a finer graded sensitivity of SCRs during the anticipation of negative events. This was done by including a larger number of different probabilities for negative events.

Three limitations of our experimental design deserve further attention. Firstly, theories of emotion guiding JDM, such as the SMH, require reafference from threat-sensitive SCRs. Without a feedback mechanism, somatic markers could not warn individuals to avoid an outcome. The underlying processes of this feedback mechanism—despite their importance for the SMH—are unclear. Further studies are needed to reveal whether such a feedback mechanism of threat-sensitive SCRs exists. While the result of our study—due to the passive nature of our experimental design– is best understood as demonstrating the coding function of SCRs, investigations of the implied feedback mechanism might help identifying causal mechanisms between decision-making processes and somatic markers.

Secondly, we reinforced negative events by means of electric shocks. As this is a specific type of negative event, it might be interesting to see whether other types of negative events, e.g. monetary losses, elicit similar physiological reactions. As mentioned earlier, it appears difficult to simulate real monetary losses in the laboratory as participants typically do not loose their “own” money [18]. One way to overcome this problem might be to provide participants with a simple task to earn money, which they lose afterwards (e.g. [29]). Some authors state that losing money that is earned should feel more like a real loss than losing money that is an initial endowment [30].

Thirdly, it is worth mentioning that the probability of receiving an electric shock and not receiving an electric shock are correlated in our experimental design. Hence, participants might consider the case of receiving an electric shock with a low probability (i.e. not receiving an electric shock with a high probability) as a positive event. Both positive and negative events might lead to an increase in SC [31]. In this case one would expect a U-shaped distribution in Fig. 3. Our results, however, show a linear increasing trend in SCRs with increasing shock probability.

Experiments applying monetary incentives may be appealing for several reasons. On the one hand, it might be interesting to identify differences between positive and negative somatic markers. Damasio points out that negative somatic markers function as alarms for an individual to avoid an outcome and positive markers function as incentives for an individual to achieve an outcome. In general, these differences can be analyzed by linking some stimuli with pleasant and some stimuli with unpleasant consequences. As there might be no positive consequences equivalent to electric shocks, monetary incentives seem to be the type of incentives necessary for this analysis. On the other hand, it is unclear how the coding of SCRs in terms of both magnitude and probability works. While both types of sensitivity seem to exist, their interaction is unclear. It would be interesting to see whether lotteries with the same expected value, but different combinations of probability and magnitude, lead to similar body reactions. Again, monetary incentives might work better than physical stimuli, as it might be difficult to find combinations of shock strength and probability which would have the same expected value. Lotteries with equivalent expected values could be played as both potential gains and potential losses. This approach may be used to study differences between positive and negative somatic markers. However, SCRs are generally insensitive towards positive and negative events, as both can lead to an increase in SC [31]. Therefore, a broader approach—including more physiological measures—appears necessary to find differences between positive and negative somatic markers.

**Fig 4:**
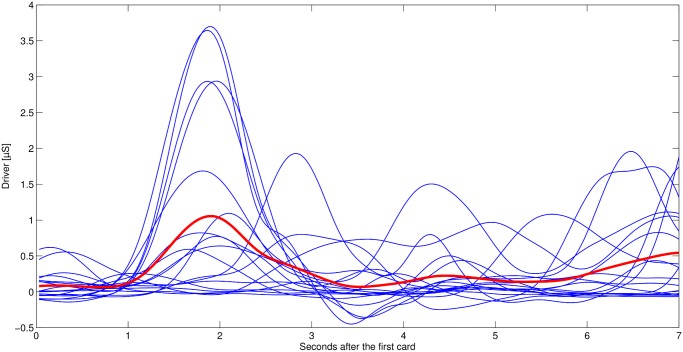
Single driver for participant 14 (50% to 39% shock probability). Red line indicates the averaged driver for these cases.

**Fig 5:**
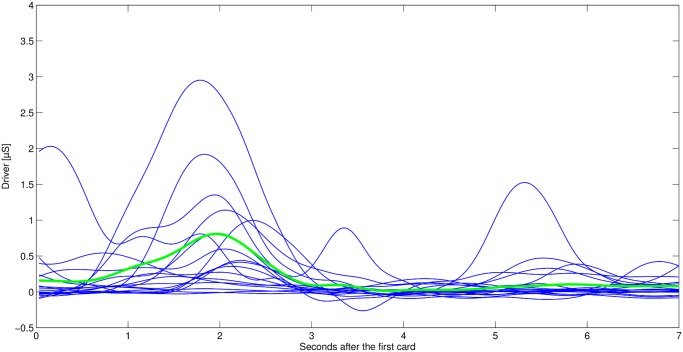
Single driver for participant 14 (0% to 11% shock probability). Green line indicates the averaged driver for these cases.

Finally, we looked at the distribution of SCRs within the time interval of interest (i.e. 2-7 s after turning the first card. SCRs within this time interval were asynchronously distributed. Fig. 4 illustrates this finding for one participant. This result is interesting because the time between the first and the second card was fixed. Therefore, participants knew that within this time interval no electric shock was to be expected. Still, SCRs indicate an increase in emotional arousal over the whole time interval in the cases with a high shock probability. By considering Figs. 4 and 5 together, it becomes visible that the aggregate increase in phasic responses in Fig. 2 does not result from a continuous increase in SC, but rather from increasingly frequent bursts.

## Conclusion

In this study, we analyze SCRs during the anticipation of unpleasant electric shocks with varying probability. Our main finding identifies a gradually increasing relation between the likelihood of unpleasant events and SCR, indicating a potential coding of probability. This result expands on previous studies that investigated the role of SCR preceding negative events of a varying magnitude. Considered together with our findings, they might give insights into the emotional processes accompanying human JDM, as SCRs seem to code the magnitude and probability of negative events. Without this sensitivity, it would be unclear how stimuli in the real world, which are typically characterized by uncertainty, are assessed in terms of emotional processes. However, the mechanism of how this information is incorporated into active decision processes requires further analysis.
